# State-Level Disparity in Lung Cancer Survival in the United States

**DOI:** 10.3389/fonc.2020.01449

**Published:** 2020-08-21

**Authors:** Yu-Che Lee, Rafael A. Calderon-Candelario, Gregory E. Holt, Michael A. Campos, Mehdi Mirsaeidi

**Affiliations:** ^1^Division of Pulmonary and Critical Care Medicine, University of Miami Miller School of Medicine, Miami, FL, United States; ^2^Section of Pulmonary Medicine, Miami Veterans Affairs Healthcare System, Miami, FL, United States; ^3^Department of Public Health Sciences, University of Miami Miller School of Medicine, Miami, FL, United States

**Keywords:** lung cancer survival, America's Health Rankings, health economy (source: MeSH NLM), insurance, disparity

## Abstract

**Rationale:** The cancer mortality-to-incidence ratio (MIR) can serve as a population-based indicator for cancer care outcomes. In the US, evaluation of lung cancer survival by individual states has not been evaluated.

**Objective:** To assess the association between lung cancer survival by using MIRs and state-level health disparities in the United States.

**Methods:** We calculated 5-year lung cancer MIR averages from 2011 to 2015 using the United States Cancer Statistics (USCS) data. America's Health Rankings (AHR) is a platform using weighted measures in five different categories to calculate annual state health rankings. Five-year averages from 2011 to 2015 of the health uninsured rate and 4-year averages from 2011 to 2014 of health spending per capita were obtained from the U.S. Census Bureau and Centers for Medicare & Medicaid Services. Linear regression analyses were performed to determine the associations between cancer survival value (CSV) = (1 – MIR) × 100% and state health variables.

**Results:** During the study period, the 5-year averages of age-adjusted incidence, mortality rates, and CSVs were 60.3 ± 2.1 per 100,000 population, 43.4 ± 2.1 per 100,000, and 27.9 ± 3.9%, respectively. Among the 50 states, Connecticut had the highest CSV (38.6 ± 1.7%) whereas Nevada had the lowest CSV (18.7 ± 6.5%). Hawaii had the highest health ranking and Mississippi had the lowest ranking in 2016. States with better health rankings, lower health uninsured rates, and higher health spending were significantly associated with higher CSVs (*R*^2^ = 0.418, *P* < 0.001; *R*^2^ = 0.352, *P* < 0.001; *R*^2^ = 0.142, *P* = 0.007, respectively).

**Conclusions:** There are significant differences in lung cancer survival within the United States. Lung cancer survival by using CSV was strongly associated with state health disparities, and it can be an applicable measure to evaluate the state-level health disparities in the United States.

## Introduction

Lung cancer is the second most commonly diagnosed cancer and the leading cause of cancer deaths in the United States (US). Approximately 228,150 new cases of lung cancer and 142,670 lung cancer deaths are expected to occur in the US in 2019 (1). However, favorable epidemiologic trends have been observed in the last decade. From 2011 to 2015, lung cancer incidence rates decreased gradually likely due to improved tobacco control and smoking cessation (2). Lung cancer mortality rates have also decreased due to reductions in smoking, improvements in early detection by low-dose spiral computed tomography (LDCT), and therapeutic advances such as improvements in minimally invasive surgery, novel chemotherapies, and biomarker-driven drugs (targeted therapies and immunotherapies) (2–4).

Racial and gender disparities in lung cancer incidence and mortality rates have been well-described, attributed not only to variations in risk factors (smoking rates) but also to variable access to screening and overall quality of care (1, 3). By estimating cancer mortality accounting for its incidence, the cancer mortality-to-incidence ratio (MIR) provides a population-based indicator of cancer survival and is a useful parameter to evaluate cancer control programs (5–7). In this way, MIR has been used to evaluate and compare differences in cancer health outcomes between health care systems worldwide, showing strong inverse correlations between cancer MIRs and the quality of health care systems (8–12). For example, for lung cancer, every 1-unit change (worsening) in health system ranking was associated with a 0.004 increment rise in MIR (9). Countries with the highest MIR included Sweden, Italy, Chile, and Estonia, while countries with lower MIR included Slovak Republic, Czech Republic, Australia, and the United States.

Despite the lower overall MIR in the US, health delivery and quality of care are not uniform as significant differences in health delivery outcomes have been described at the state level in terms of health uninsured rates and health spending per capita (13). We hypothesize that state variation in lung cancer MIR will be correlated with state's health rankings because of prior cross-country studies (8–12), with the health uninsured rate because of lower use of preventive screenings and later stage diagnoses, and with health spending per capita because of lower use of expensive new curative procedures. Therefore, the aim of this study was to analyze the association of these variable state health delivery parameters and lung cancer outcomes by using MIRs to provide another perspective of the impact of state-level health disparities in the United States.

## Materials and Methods

### Data Sources

Age-adjusted mortality and incidence rates per 100,000 population per year were obtained from the United States Cancer Statistics (USCS) database provided by the Centers for Disease Control and Prevention (CDC). MIR calculations were performed by dividing the age-adjusted mortality rate by the age-adjusted incidence rate per 100,000. With these data, we calculated 5-year averages from 2011 to 2015 for all 50 states. We defined the statistic, cancer survival value (CSV) as (1 – MIR) × 100% where values approaching 0% represent a poor survival rate and those approaching 100% represent an excellent survival rate. America's Health Rankings (AHR) was created as a partnership between the United Health Foundation and the American Public Health Association. AHR evaluates the factors that influence health outcomes and determine the state's health rankings since 1990. The numerical health ranking was calculated for each state in the year 2016 by an equation using weighted measures in five different categories: 25% for Behaviors, 22.5% for Community & Environment, 12.5% for Policy, 15% for Clinical Care, and 25% for Outcomes. In addition, 5-year averages of health uninsured rate by state from 2011 to 2015 were obtained from the U.S. Census Bureau and 4-year averages of health spending per capita by state from 2011 to 2014 were obtained from Centers for Medicare & Medicaid Services.

### Statistical Analyses

Scatterplots of state health rankings, health uninsured rates by state, and health spending per capita by state vs. CSV were generated, respectively. The association between CSVs and state health variables was calculated by three separate univariate linear regression analyses with the following formulas: CSV = State Health Ranking ^*^ Beta + Alpha; CSV = Health Uninsured Rates ^*^ Beta + Alpha; CSV = Health Spending per capita ^*^ Beta + Alpha. All data management and statistical analyses were performed in Microsoft Excel and Statistics Software SAS^®^. *P* < 0.05 using two-sided *t* tests were considered statistically significant.

## Results

### Lung Cancer Incidence, Mortality Rates, and CSVs by State

**Figure 1:**
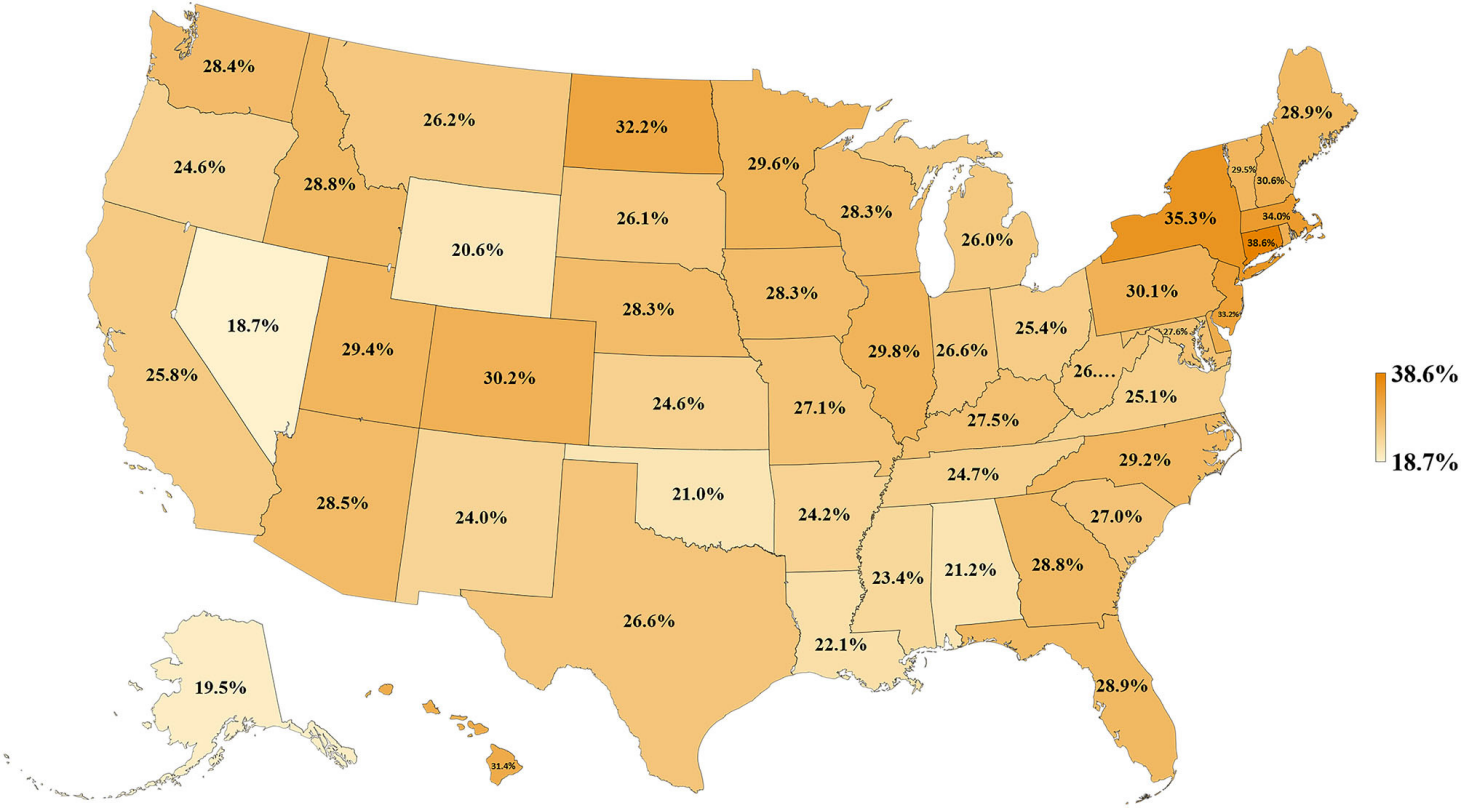
Map of lung cancer CSVs for 50 states of the US, 2011–2015.

**Figure 2:**
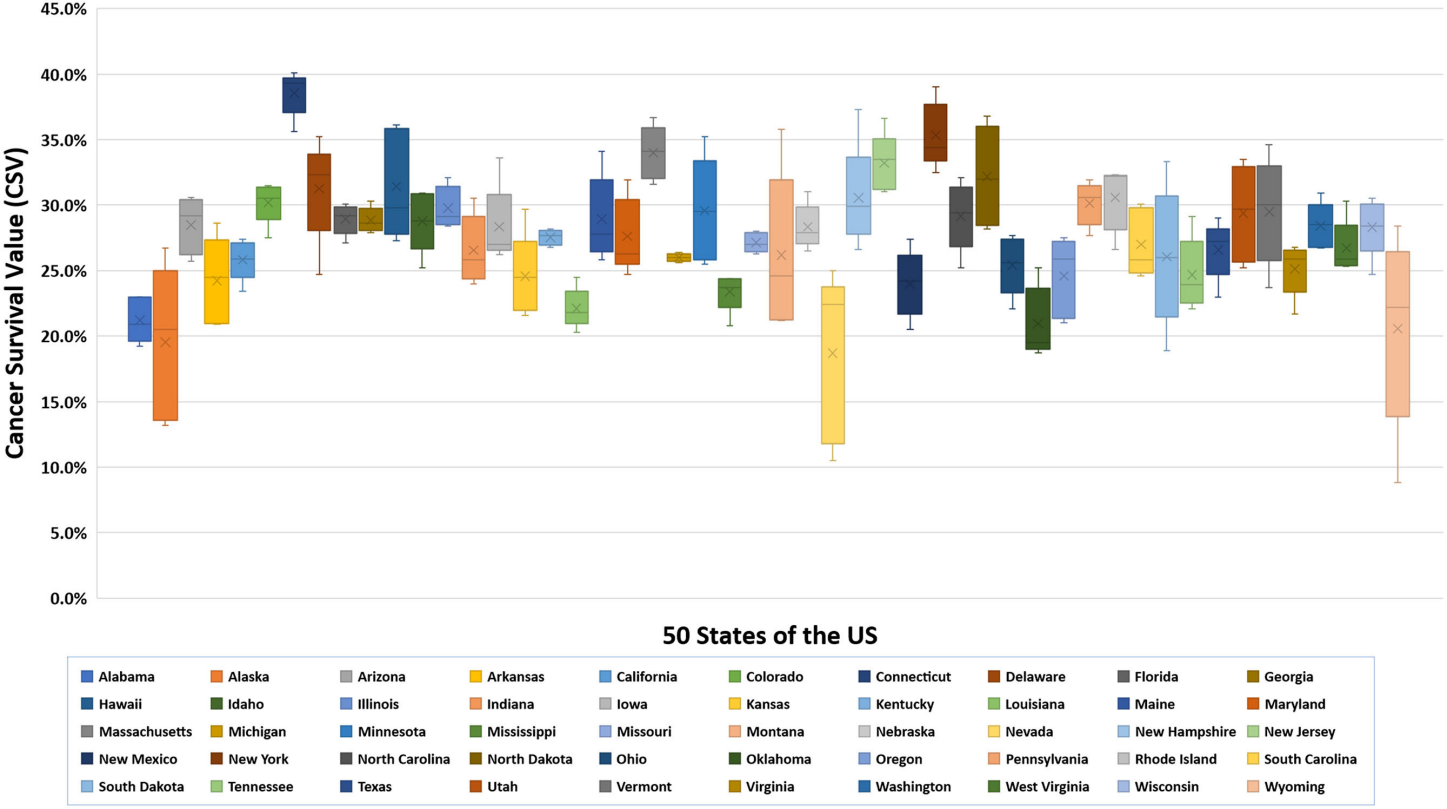
Boxplot of lung cancer CSVs for 50 states of the US, 2011–2015.

We first examined lung cancer statistics in the United States by analyzing the average age-adjusted incidence, mortality rates, and CSVs from 2011 to 2015. During this period, a total of 1,087,810 people were diagnosed with lung cancer and 779,796 people died from lung cancer. The overall 5-year average of age-adjusted incidence, mortality rates, and CSVs were 60.3 ± 2.1 per 100,000 population, 43.4 ± 2.1 per 100,000, and 27.9 ± 3.9%, respectively. The analysis based on 50 states indicated that Kentucky had the highest age-adjusted incidence and mortality rate (93.5 ± 2.4 and 67.8 ± 2.2 per 100,000, respectively), whereas Utah had the lowest age-adjusted incidence and mortality rate (27.7 ± 2.4 and 19.5 ± 0.7 per 100,000, respectively). Regarding CSVs, Connecticut had the highest CSV (38.6 ± 1.7%) and Nevada had the lowest CSV (18.7 ± 6.5%). The results are summarized in Table 1 and shown in Figures 1, 2.

**Table 1:** Summary of state health rankings, age-adjusted incidence and mortality rate, cancer survival value (CSV) for lung cancer, health uninsured rate, and health spending per capita of 50 states.

**State**	**2016 America's Health Rankings (AHR)**	**2011–2015** **Five-Year Average of** **Age-Adjusted Rate per 100,000**		**2011–2015** **Cancer Survival Value (%)**		**2011–2015** **Five-Year Average of Health Uninsured Percentage (Standard Deviation)**	**2011–2014** **Four-Year Average of Health Spending Per Capita, Dollars per Year** **(Standard Deviation)**
		**Incidence** **(Standard Deviation)**	**Mortality** **(Standard Deviation)**	**Mean** **(Standard Deviation)**	**Median** **(Interquartile Range)**		
Hawaii	1	46.2 (2.5)	31.6 (1.5)	31.4 (4.1)	29.8 (27.8–35.9)	6.0 (1.3)	6,896.0 (321.8)
Massachusetts	2	63.8 (1.9)	42.1 (2.5)	34.0 (2.0)	34.1 (32.1–35.9)	3.6 (0.6)	10,180.3 (313.7)
Connecticut	3	61.0 (2.5)	37.5 (2.1)	38.6 (1.7)	39.3 (37.1–39.7)	8.0 (1.5)	9,406.5 (381.5)
Minnesota	4	55.2 (1.9)	38.8 (1.0)	29.6 (4.0)	29.5 (25.8–33.4)	7.1 (1.8)	8,370.3 (391.1)
Vermont	5	63.5 (3.1)	44.8 (3.6)	29.5 (4.1)	30.0 (25.8–33.0)	5.8 (1.4)	9,571.3 (595.1)
New Hampshire	6	65.8 (3.4)	45.7 (3.9)	30.6 (4.0)	29.9 (27.8–33.7)	9.5 (1.9)	9,193.0 (361.1)
Washington	7	56.9 (3.1)	40.7 (2.4)	28.4 (1.7)	28.5 (26.8–30.0)	11.6 (3.5)	7,525.5 (329.9)
Utah	8	27.7 (2.4)	19.5 (0.7)	29.4 (3.7)	29.7 (25.7–33.0)	13.4 (1.9)	5,631.0 (268.2)
New Jersey	9	57.4 (1.8)	38.4 (2.4)	33.2 (2.2)	33.5 (31.2–35.1)	11.7 (1.9)	8,379.8 (380.1)
Colorado	10	43.4 (2.1)	30.3 (2.1)	30.2 (1.6)	30.5 (28.9–31.4)	12.5 (3.1)	6,427.8 (287.3)
North Dakota	11	58.3 (2.4)	39.6 (3.2)	32.2 (3.8)	32.0 (28.5–36.0)	9.2 (1.2)	9,242.8 (481.6)
Nebraska	12	59.0 (2.2)	42.3 (0.9)	28.3 (1.7)	27.9 (27.1–29.9)	10.4 (1.4)	8,059.8 (291.4)
New York	13	60.2 (1.7)	39.0 (2.3)	35.3 (2.5)	34.4 (33.4–37.7)	9.8 (1.8)	9,305.3 (347.3)
Rhode Island	14	69.6 (2.5)	48.3 (1.3)	30.6 (2.5)	32.2 (28.1–32.3)	9.3 (2.6)	9,113.5 (330.0)
Idaho	15	50.8 (1.8)	36.2 (1.2)	28.8 (2.3)	28.8 (26.7–30.9)	14.7 (2.4)	6,508.8 (335.8)
California	16	43.4 (1.8)	32.2 (1.9)	25.8 (1.5)	25.9 (24.5–27.1)	14.8 (4.2)	7,140.0 (345.5)
Iowa	17	63.6 (0.7)	45.6 (1.6)	28.3 (3.0)	27.0 (26.6–30.8)	7.3 (1.7)	7,767.5 (329.8)
Maryland	18	57.5 (1.6)	41.6 (2.5)	27.6 (2.8)	26.3 (25.5–30.4)	9.1 (1.7)	8,226.0 (281.5)
Virginia	19	58.9 (2.8)	44.1 (2.6)	25.1 (2.0)	25.9 (23.4–26.6)	11.5 (1.5)	7,221.0 (284.9)
Wisconsin	20	60.0 (2.0)	43.0 (2.7)	28.3 (2.2)	28.4 (26.5–30.1)	8.0 (1.5)	8,230.8 (326.7)
Oregon	21	56.4 (4.9)	42.5 (3.2)	24.6 (3.0)	25.9 (21.4–27.2)	12.4 (3.8)	7,400.3 (476.9)
Maine	22	72.6 (2.9)	51.6 (2.8)	28.9 (3.2)	27.8 (26.5–32.0)	10.1 (1.1)	9,122.3 (300.5)
Montana	23	55.8 (3.6)	41.2 (4.0)	26.2 (6.1)	24.6 (21.3–31.9)	15.7 (2.8)	7,790.3 (403.1)
South Dakota	24	58.1 (2.0)	42.9 (2.7)	26.1 (5.3)	26.0 (21.5–30.7)	10.9 (0.9)	8,470.0 (366.3)
Wyoming	25	44.6 (3.7)	35.3 (2.9)	20.6 (7.4)	22.2 (13.9–26.5)	13.5 (1.8)	7,917.0 (317.9)
Illinois	26	66.1 (1.9)	46.4 (2.0)	29.8 (1.6)	29.1 (28.5–31.4)	11.1 (2.6)	7,816.8 (356.1)
Kansas	27	60.9 (1.9)	45.9 (1.5)	24.6 (3.2)	24.5 (22.0–27.2)	11.4 (1.6)	7,427.3 (206.8)
Pennsylvania	28	64.7 (1.6)	45.3 (1.9)	30.1 (1.6)	30.6 (28.5–31.5)	8.9 (1.5)	8,799.8 (355.6)
Arizona	29	49.5 (2.5)	35.4 (1.6)	28.5 (2.2)	29.2 (26.2–30.4)	15.3 (3.0)	6,243.3 (158.7)
Alaska	30	57.5 (4.7)	46.2 (4.6)	19.5 (5.9)	20.5 (13.6–25.0)	18.2 (2.3)	10,302.0 (616.1)
Delaware	31	71.2 (3.1)	48.9 (3.1)	31.2 (3.9)	32.3 (28.1–33.9)	8.2 (1.4)	9,666.5 (452.9)
North Carolina	32	69.3 (1.4)	49.1 (2.5)	29.2 (2.6)	29.4 (26.9–31.4)	14.6 (2.3)	7,043.0 (187.3)
Texas	33	53.2 (2.6)	39.1 (2.9)	26.6 (2.2)	27.2 (24.7–28.2)	20.8 (2.5)	6,636.5 (278.4)
Michigan	34	65.6 (2.5)	48.6 (1.9)	26.0 (0.3)	26.0 (25.7–26.3)	9.8 (2.4)	7,710.8 (269.6)
Nevada	35	56.4 (6.7)	45.5 (2.5)	18.7 (6.5)	22.4 (11.8–23.8)	18.5 (4.5)	6,243.5 (341.9)
Florida	36	59.8 (3.5)	42.5 (2.1)	28.9 (1.2)	29.2 (27.9–29.9)	18.2 (3.2)	7,701.8 (277.5)
Missouri	37	74.3 (2.4)	54.1 (1.9)	27.1 (0.7)	27.0 (26.5–27.9)	12.4 (1.6)	7,791.5 (275.8)
New Mexico	38	40.3 (1.6)	30.6 (1.2)	24.0 (2.5)	24.2 (21.7–26.2)	16.4 (3.7)	6,853.0 (270.1)
Indiana	39	72.9 (1.6)	53.5 (1.9)	26.6 (2.6)	25.8 (24.4–29.1)	12.9 (2.1)	7,839.3 (418.5)
Ohio	40	69.4 (1.9)	51.8 (2.5)	25.4 (2.2)	25.6 (23.3–27.4)	9.9 (2.3)	8,180.5 (441.5)
Georgia	41	65.1 (3.0)	46.3 (2.8)	28.8 (1.0)	28.6 (28.1–29.8)	17.3 (2.4)	6,139.5 (370.2)
South Carolina	42	67.0 (2.0)	48.9 (3.1)	27.0 (2.6)	25.8 (24.8–29.8)	14.8 (2.5)	6,972.8 (259.2)
West Virginia	43	80.3 (2.2)	58.8 (2.0)	26.7 (2.1)	25.9 (25.4–28.5)	11.6 (4.0)	8,865.8 (494.6)
Tennessee	44	75.8 (0.8)	57.0 (2.0)	24.7 (2.8)	23.9 (22.5–27.3)	12.9 (1.8)	7,056.8 (260.4)
Kentucky	45	93.5 (2.4)	67.8 (2.2)	27.5 (0.6)	27.7 (27.0–28.1)	11.4 (3.9)	7,494.5 (377.9)
Oklahoma	46	70.5 (2.3)	55.7 (2.5)	21.0 (2.7)	19.5 (19.0–23.7)	16.8 (2.1)	7,254.0 (293.1)
Alabama	47	67.8 (3.3)	53.5 (2.9)	21.2 (1.7)	20.9 (19.6–23.0)	12.7 (1.6)	6,909.8 (310.4)
Arkansas	48	78.0 (1.4)	59.1 (2.3)	24.2 (3.3)	24.5 (21.0–27.4)	14.2 (3.3)	6,931.5 (348.0)
Louisiana	49	68.9 (2.4)	53.7 (2.5)	22.1 (1.5)	21.8 (21.0–23.4)	15.5 (2.3)	7,441.5 (282.5)
Mississippi	50	75.0 (1.7)	57.5 (1.9)	23.4 (1.5)	23.7 (22.2–24.4)	15.8 (2.1)	7,302.5 (339.8)

### State Health Rankings, Health Uninsured Rate, and Health Spending per Capita Were Significantly Associated With Lung Cancer CSVs

**Figure 3:**
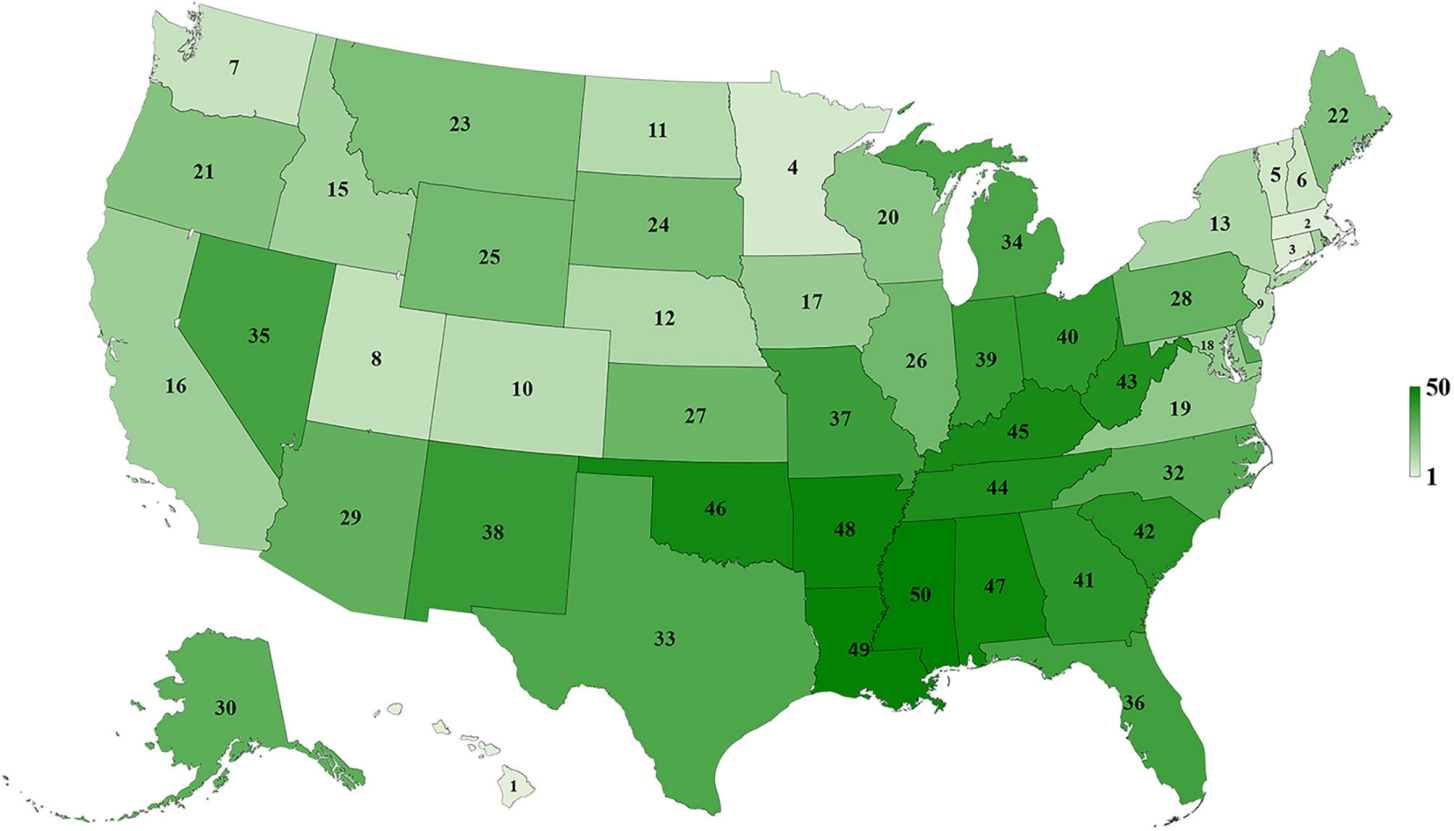
Map of 2016 state health rankings for 50 states of the US.

**Figure 4:**
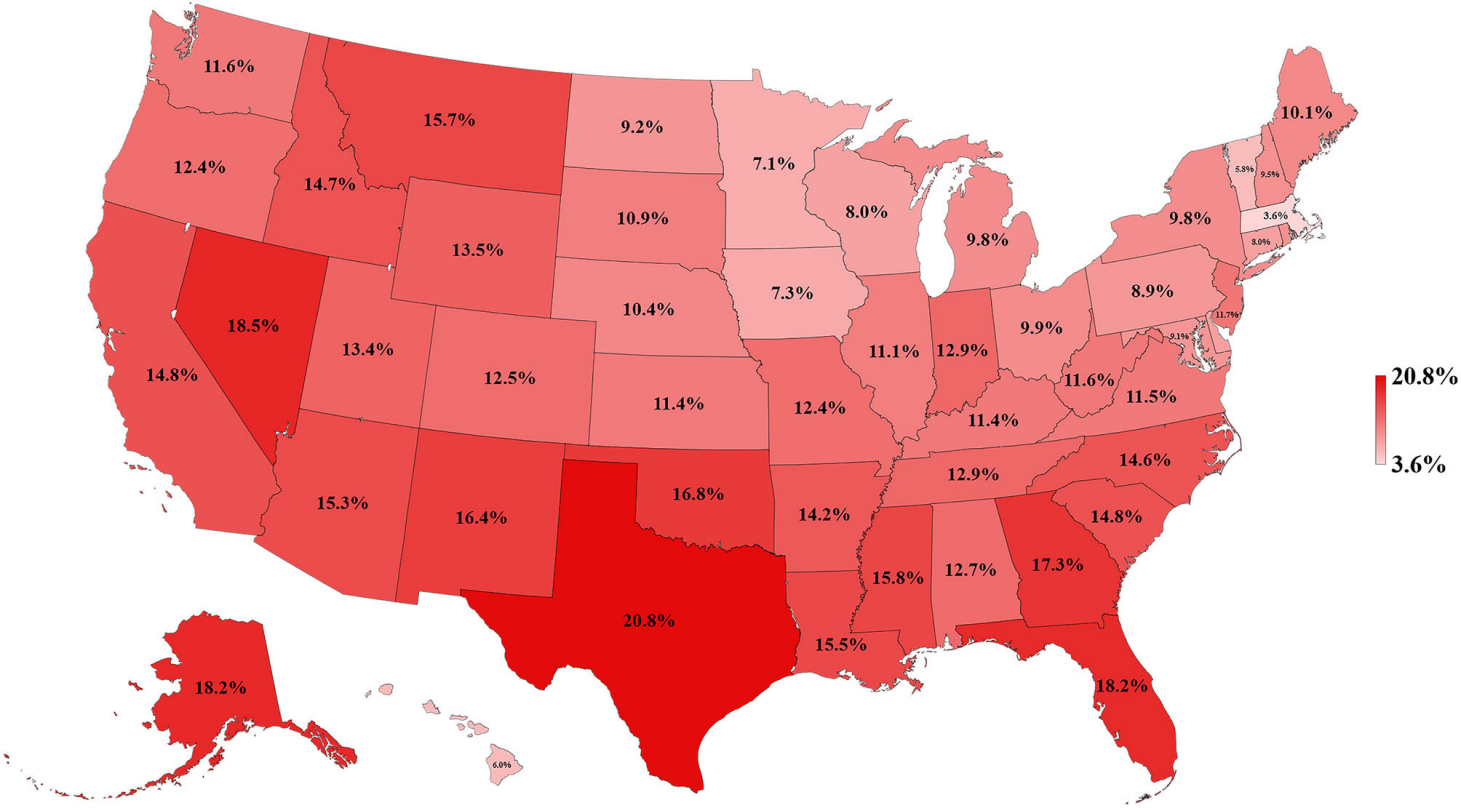
Map of health uninsured rate for 50 states of the US, 2011–2015.

**Figure 5:**
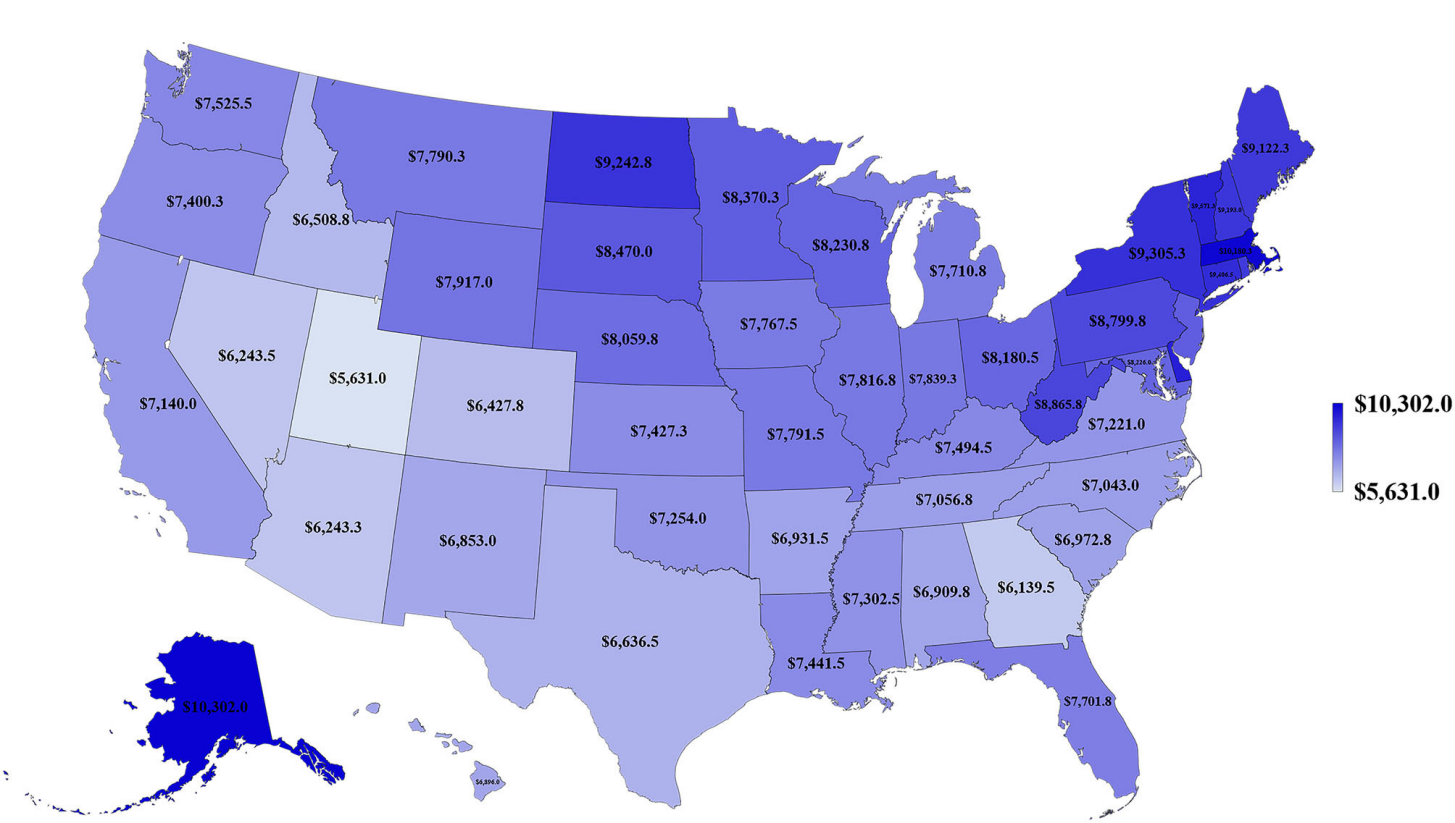
Map of health spending per capita for 50 states of the US, 2010–2014.

**Figure 6:**
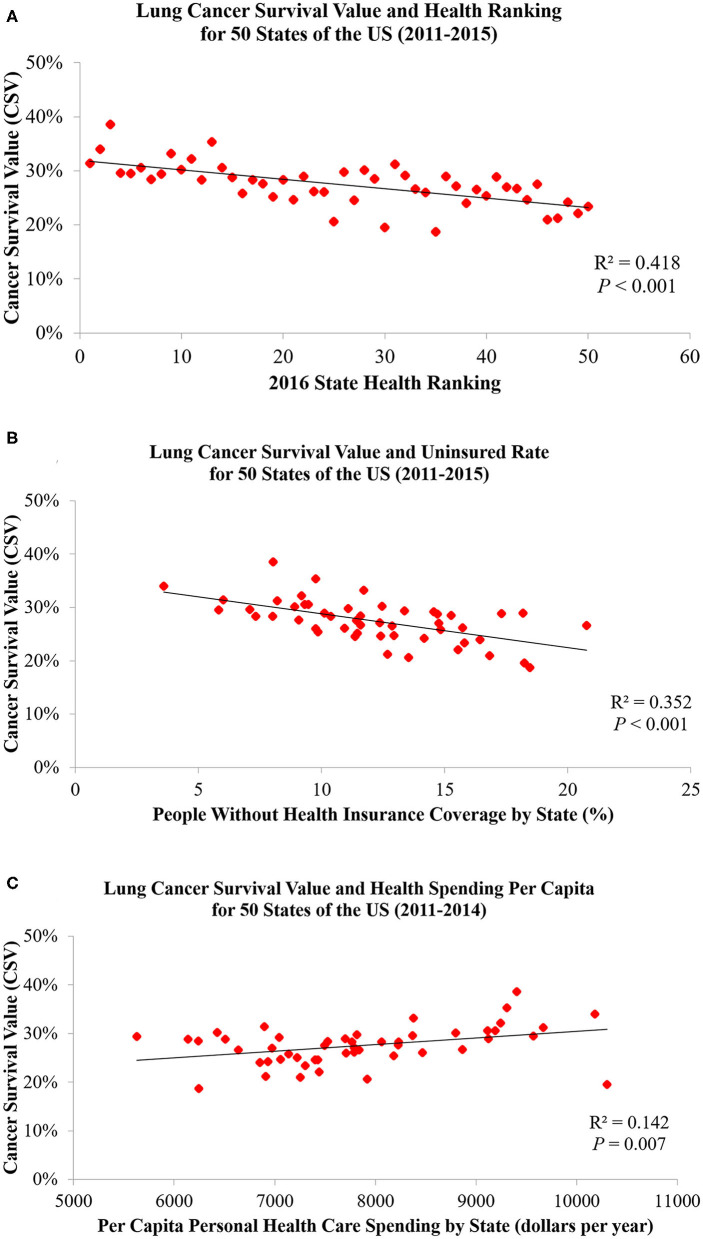
Lung cancer CSVs are significantly associated with **(A)** state health rankings, **(B)** health uninsured rates, and **(C)** health spending per capita.

Table 1 and Figures 3–5 show the differences in health delivery outcomes for the 50 states using the 2016 state health rankings, the 5-year average (2011–2015) of health uninsured rates per state, and the 4-year average (2011–2014) of health spending per capita per state. In regard to state health rankings, Hawaii had the highest health ranking and Mississippi had the lowest health ranking, and it is significantly associated with improved CSV (*R*^2^ = 0.418, *P* < 0.001, Figure 6A). The regression formula is *y* = −0.0017*x* + 0.3194, wherein, for every 1-unit change (worsening) in health system ranking, there is a 0.0017 decrement in the CSV. For health uninsured rate, the highest was Texas (20.8 ± 2.5%) and the lowest was Massachusetts (3.6 ± 0.6%), and it is negatively correlated with CSV (*R*^2^ = 0.352, *P* < 0.001, Figure 6B). Massachusetts also had the second highest health spending per capita ($10,180.3 ± 313.7 per year) and Utah had the lowest health spending per capita ($5,631.0 ± 268.2 per year). Health spending is also associated with CSV (*R*^2^ = 0.142, *P* = 0.007, Figure 6C).

## Discussion

To our knowledge, this is the first study to evaluate the association between lung cancer CSVs and state health delivery variables within the US. This study demonstrates clear geographic variations in lung cancer care outcome rates between states and its marked association with state-level health disparities. The CSV, derived from the validated MIR, is regarded as a valid proxy for 5-year relative survival and an indicator of cancer care delivery outcomes (14, 15). Although Ellis et al. recently suggested that MIR should not be used as a proxy of cancer survival in England, their study showed the absolute difference between the CSV and 5-year survival was <10% for lung cancer, which is similar to the previous study result in the United States, the Netherlands, and the five Nordic countries (15, 16). Therefore, CSV could still be considered as a good approximation of the 5-year relative survival for lung cancer but not all tumor sites. The mapping of lung cancer CSVs shows that the Northeastern states have higher relative lung cancer survival whereas Southern states have lower relative lung cancer survival. Mortality is influenced by early interventions in risk factor reduction (tobacco smoking), early detection with LDCT among smokers, and effective application of state-of-the-art advances in treatment including surgery, focused radiotherapy chemotherapy, targeted drugs, and/or immunotherapy for lung cancer (2–4, 17–21). The variability in health delivery per state, namely, health rankings, uninsured rates, and total health care spending per capita, may reflect variable access to these preventive and therapeutic options leading to clear variability in lung cancer CSV within the country. In our model, total health ranking had the highest *R*^2^ explaining 42% of the variability of CSV, followed by uninsured rates (35%) and health spending per capita (14%).

The Northeastern states have higher relative lung cancer survival, better health rankings, lower health uninsured rate, and higher health spending per capita compared to the Southern states. This phenomenon can be explained by the similar characteristics among adjacent states, which include the demographics [percentage of total African American population by region according to the 2010 Census: Northwest (17%), Midwest (18.1%), South (55.0%), and West (9.8%)], numbers of physicians per capita, socioeconomic conditions, the availability of medical services, incomes of residents, and the levels of benefits provided by public and private health programs (22–27). Affordability and access to state-of-the art cancer care are important factors for geographic differences of cancer survival. Interestingly, Onega et al. indicated the Northeastern states had the shortest travel times to National Cancer Institute (NCI) Cancer Centers and the highest per capita oncologist supply, followed by the Western, the Midwestern, and then the Southern states (28). This suggested that a better geographic access to cancer care is linked to a significant cancer survival benefit (29).

We found a negative correlation between state health rankings and lung cancer CSVs that is similar to a previous study result on colorectal cancer among the Organization for Economic Co-operation and Development (OECD) countries (11). Sunkara et al. identified a strong relationship between colorectal cancer MIRs and WHO rankings of OECD countries, which suggests that better colorectal cancer survival is highly associated with better quality of cancer screening and health care systems. Similarly, Choi et al. found an important correlation between lung cancer MIR and health care rankings between OECD countries with an *R*^2^ of 0.32 and 0.49 after removing outliers (9). Therefore, our findings may be a reflection of the profound impact that health policies and delivery at a state level have on clinical outcomes. Early detection and appropriate treatments for lung cancer, including surgical interventions and advanced therapies, lead to large health expenditures (30–32). For example, Vera-Llonch et al. in their study, indicated that health care costs among patients with metastatic lung cancer receiving chemotherapy are substantial, exceeding $125,000 per patient over a mean follow-up period of 500 days. This might be the reason that lung cancer CSVs are significantly associated with health uninsured rates and health spending per capita (2).

There are some limitations to our study. First, no further clinical information including lung cancer types, stages at diagnosis, molecular characteristics, or application of LDCT screening was available to create a comprehensive model. Second, the use of the state health rankings, health uninsured rates, and health spending per capita to represent the health disparities of states are not the only parameters to evaluate the status of health care delivery. We did not have access to the lung cancer risk factors rates, state-specific prevention programs, and other socioeconomic factors of relevance, which may play crucial roles in explaining the incidence and mortality rates. As a result, further investigations with greater detail and data are needed to support our findings.

In conclusion, this study showed that lung cancer CSVs are significantly associated with state health rankings, health uninsured rates, and health spending per capita. These findings suggest that CSVs of lung cancer can be an applicable measure to evaluate and reflect the state-level health disparities in the United States. Our study also provides evidence for policy makers and public health practitioners developing more effective prevention and interventions for lung cancer.

## Data Availability Statement

The raw data supporting the conclusions of this article will be made available by the authors, without undue reservation.

## Author Contributions

Y-CL had full access to all of the data in the study and takes responsibility for the integrity of the data and the accuracy of the data analysis. Y-CL and MM contributed to the literature review, study concept and design, data analysis, and interpretation. Y-CL, RC-C, GH, MC, and MM contributed to the writing and revision of the manuscript. All authors contributed to the article and approved the submitted version.

## Conflict of Interest

The authors declare that the research was conducted in the absence of any commercial or financial relationships that could be construed as a potential conflict of interest.

## Abbreviations

AHR: America's Health Rankings
CSV: cancer survival value
LDCT: low-dose spiral computed tomography
MIR: mortality-to-incidence ratio
OECD: Organization for Economic Co-operation and Development.
